# Supplementary material for: From sequence to enzyme mechanism using multi-label machine learning
Source: BMC Bioinformatics. 2014 May 19;15:150. doi: 10.1186/1471-2105-15-150 (PMC4229970; doi:10.1186/1471-2105-15-150)
Supplement: Additional file 2 — Java code of ml2db. Additional file ml2db_code.tar.gz contains the Java source code to run the multi-label machine learning experiments and save the results to database. The code’s Javadoc is included. [file 1471-2105-15-150-S2.zip › additional file 2/ml2db/utils/doc/test/package-tree.html]

test Class Hierarchy


---


|  |  |  |
| --- | --- | --- |
| **Overview** \| **Package** \| Class \| Use \| **Tree** \| **Deprecated** \| **Index** \| **Help** | |  |
| **PREV**   **NEXT** | **FRAMES**    **NO FRAMES**     **All Classes** |  |


---


## Hierarchy For Package test

**Package Hierarchies:**: All Packages

---

## Class Hierarchy

- java.lang.Object
  - test.**AllDatabaseUtilsTests**- test.**AllUtilsTests**- junit.framework.Assert
        - junit.framework.TestCase (implements junit.framework.Test)
          - test.**CollectionUtilsTest**- test.**Data**- test.**ListUtilsTest**- test.**NumberUtilsTest**- test.**ReflectionUtilsTest**- test.**RegExpUtilsTest**- test.**SimpleDOMParserTest**- test.**StringUtilsTest**- test.**TimeUtilsTest**- test.**UtilsTest**- test.**WebUtilsTest**- test.**XmlNodeTest**- test.**XmlSearcherTest**- test.**XmlUtilsTest**
        - test.**FileUtilsTest**

---


|  |  |  |
| --- | --- | --- |
| **Overview** \| **Package** \| Class \| Use \| **Tree** \| **Deprecated** \| **Index** \| **Help** | |  |
| **PREV**   **NEXT** | **FRAMES**    **NO FRAMES**     **All Classes** |  |


---
